# Genetic variation and genome-wide association analysis of nitrogen use efficiency-related traits under combined heat and nitrogen-deficient stress in an *Aegilops tauschii*-derived wheat population

**DOI:** 10.3389/fpls.2025.1621916

**Published:** 2025-07-16

**Authors:** Amir Ibrahim Ismail Emam, Izzat Sidahmed Ali Tahir, Nasrein Mohamed Kamal, Yasir Serag Alnor Gorafi, Hisashi Tsujimoto, Takayoshi Ishii

**Affiliations:** ^1^ United Graduate School of Agricultural Sciences, Tottori University, Tottori, Japan; ^2^ Wheat Research Program, Agricultural Research Corporation, Wad Medani, Sudan; ^3^ International Platform for Dryland Research and Education (IPDRE), Tottori University, Tottori, Japan; ^4^ Graduate School of Agriculture, Kyoto University, Kyoto, Japan; ^5^ Arid Land Research Center (ALRC), Tottori University, Tottori, Japan; ^6^ Chromosome Engineering Research Center, Tottori University, Yonago, Japan

**Keywords:** heat stress, genetic diversity, MTAs, nitrogen deficiency, nitrogen uptake efficiency, relative performance, stress tolerance index, wild relatives

## Abstract

Heat stress and nitrogen (N) deficiency increasingly limit global wheat (*Triticum aestivum* L.) yields, highlighting the need to improve nitrogen use efficiency (NUE) under combined stresses for sustainable production. We assessed 145 multiple-synthetic-derivative (MSD) lines, carrying alleles from diverse *Ae. tauschii*, crossed and backcrossed into ‘Norin 61’, together with three checks across six field environments combining heat stress and either optimal (86 kg N ha^-^¹; HS-HN) or zero (HS-LN) N supply in central Sudan. Eighteen agronomic and physiological traits were recorded, and best linear unbiased estimates were used for genome-wide association analysis (GWAS) with 31,362 high-quality DArTseq and GRAS-Di markers. HS-LN reduced mean grain yield (GY) and grain N uptake (GNUp) by 14% and 28%, respectively, but increased thousand-kernel weight and harvest index, indicating resource re-allocation to grain filling. The MSD lines showed wide variation, and some lines maintained high GY under either HS-HN (e.g., MSD053 and MSD450) or HS-LN (e.g., MSD192 and MSD383). The MSD lines MSD026, MSD181, and MSD485 ranked among the top five for GY under HS-LN, HS-HN conditions, and across the six environments. GWAS identified 34 marker-trait associations (MTAs) on 12 chromosomes; 62% resided in the D subgenome. A pleiotropic locus on 5A (rs987242) affected grain growth rate and GY, whereas a novel locus on 3D (rs1071033) explained 88% of the variation in GNUp relative performance. Candidate genes included mitogen-activated protein kinases, DELLA (Rht-1), MADS-box, and DnaJ homologues linked to stress signaling or N metabolism. Our results uncover genetic variants and germplasm that enhance NUE and yield stability under concurrent heat and N stress, providing immediately deployable resources for climate-resilient wheat breeding.

## 1 Introduction

Wheat (*Triticum aestivum* L.) is one of the five major cereal crops, grown on approximately 220 million hectares and producing approximately 799 million tons annually (FAOSTAT, 2023). The significant increase in wheat productivity in recent decades, primarily driven by the Green Revolution, has been instrumental in addressing global food security through the development of high-yielding semi-dwarf, fertilizer-responsive varieties (Asami, 2023). Among fertilizers, nitrogen (N) represents the most extensively used fertilizer, with about 111.6 million tons used globally (FAO, 2022). Nitrogen deficiency is a major constraint that limits wheat yield and quality (Wang et al., 2021). Insufficient nitrogen supply severely affects plants, resulting in leaf chlorosis, stunted growth, decreased plant height, fewer tillers and grain numbers, reduced biomass, and consequently decreased yield (Shirazi et al., 2014; Das et al., 2023). Although increasing N fertilizer application has been a common strategy to enhance both yield and quality, only approximately 30% of the applied nitrogen is efficiently utilized by plants, indicating excessive loss to the environment through volatilization, leaching, and runoff, leading to serious environmental concerns and significant economic costs (Garnett et al., 2015; Das et al., 2023; Govindasamy et al., 2023). Moreover, nitrogen fertilizers are considered a major source of greenhouse gas nitrous oxide, with about 10.6% of the emissions coming from agricultural fields (Menegat et al., 2022). Therefore, maintaining yield increase following the green revolution model under the current global challenges of climate change and resource depletion is no longer a strategic and environmentally friendly option for humanity.

Building resilient and sustainable agricultural systems is key to achieving sustainable human life, including a healthy and productive environment. These sustainable agricultural systems need high-yielding crops with enhanced resource utilization efficiency, such as water and fertilizers. In this regard, enhancing nitrogen use efficiency (NUE) is essential for achieving sustainable wheat production (Barraclough et al., 2010; Cormier et al., 2016; Tahir et al., 2020; Salim and Raza, 2020). Genetic and agronomic strategies provide effective solutions for improving NUE while mitigating the environmental impact of excessive fertilizer use. Genetic approaches focus on identifying and incorporating alleles associated with improved NUE in breeding programs, while agronomic interventions emphasize precision fertilizer application, optimized timing, and integrated nutrient management systems (Foulkes et al., 2009; Cormier et al., 2013; Guttieri et al., 2017).

Due to climate change, global temperatures are expected to increase, and wheat is expected to experience warmer temperatures than usual in environments currently considered optimum production areas. In addition, due to increased consumption resulting from dietary changes, wheat is expanding in warmer marginal areas. Extensive research has been conducted on wheat response to nitrogen deficiency, NUE, and heat stress tolerance as independent factors (Hussain et al., 2006; Zhang et al., 2017; Riaz et al., 2021; Mahdavi et al., 2022; Mini et al., 2023; Hu et al., 2024). However, the combined effects of these stresses remain poorly understood (Modhej et al., 2008; Liu et al., 2013; Elía et al., 2018). Therefore, addressing this critical knowledge gap is essential because simultaneous exposure to nitrogen deficiency and heat stress can trigger unique physiological and molecular responses distinct from those observed under individual stress conditions (Dixit et al., 2024). High temperature reduces photosynthesis, damages chloroplasts, and causes leaf senescence, which limits nutrient uptake and translocation, especially N, and thus reduces the positive yield response to applied nitrogen (Tahir and Nakata, 2005; Liu et al., 2013; Akter and Rafiqul Islam, 2017). Nevertheless, genetic gain in wheat NUE has been reported under heat stress; however, further improvement in NUE may require broadening the genetic diversity (Tahir et al., 2020).

The modern wheat crop is known for its narrow genetic variability, resulting from its evolution, domestication, centuries of cultivation, and extensive modern breeding and selection (Venske et al., 2019). Wheat has tremendous genetic diversity in wild relatives adapted to harsh environments and possesses genes and alleles that can improve cultivated wheat resilience. Among these wild relatives, *Ae. tauschii* is considered an attractive source of genes to enhance wheat biotic and abiotic stress tolerance. Gorafi et al. (2018) proposed a multiple synthetic derivative (MSD) population as a platform for harnessing *Ae. tauschii* genetic diversity for wheat improvement; since then, several studies have used the MSD lines and identified germplasm lines and marker-trait associations for heat and drought stress tolerance, grain shape and hardness, bread making, and nutrient quality (Elhadi et al., 2021b, a; Itam et al., 2022; Mohamed et al., 2022; Emam et al., 2025). Therefore, we investigated the response of 145 MSD lines to combined heat-nitrogen deficiency stress to (1) identify MSD lines with enhanced NUE, (2) identify marker-trait associations (MTAs) associated with traits related to NUE, and (3) identify novel alleles derived from *Ae. tauschii* for potential use as a key source to improve NUE in wheat under combined heat-nitrogen stress.

## 2 Materials and methods

### 2.1 Plant materials

The plant material comprised 148 wheat genotypes, including 145 multiple synthetic derivative (MSD) lines, their recurrent parent Norin 61 (N61), and two Sudanese heat-stress adapted cultivars, Imam and Goumria. The MSD population was created by crossing and backcrossing 43 synthetic hexaploid wheat lines with N61, a standard Japanese cultivar, that generated a population of 400 lines. The 43 synthetic hexaploid lines were derived from crosses between *Triticum turgidum* cv. durum Langdon and *Ae. tauschii* accessions from diverse sources covering the entire species range and taxonomical lineages (Matsuoka et al., 2007; Gorafi et al., 2018, 2025). From the initial 400 lines, excluding all lines with more than 75 days to heading, a more homogeneous population of 145 MSD lines was evaluated under the heat stress (HS) conditions at Wad Medani, Sudan, combined with either the recommended nitrogen dose (HS-HN) or a low nitrogen level (HS-LN).

### 2.2 Experimental conditions

All experiments were conducted at the Gezira Research Farm, Agricultural Research Corporation, Wad Medani, Sudan (14°24′N, 29°33′E, 407 m.a.s.l.). Wad Medani is classified as a hot and dry wheat-growing area (Rajaram et al., 1994). The soil at Wad Medani is classified as a typic haplustert soil, with a heavy clay texture that swells upon wetting and shrinks when dry, and a pH of 8.0-8.5. It contained low levels of organic matter, nitrogen, and available phosphorus. The soil in the experimental plots had a nitrogen content range of 0.025-0.03% in the top 0-60 cm, whereas the phosphorus content was in the range of 3.0-5.4 mg/kg soil.

Experiments were conducted during the 2018/19, 2019/20, and 2020/21 seasons. All experiments were performed using an alpha-lattice design with two replications. Each genotype was assigned to a plot of four rows, 1 m long and spaced 0.2 m. Seeds were treated with insecticide (Gaucho, imidacloprid, 35% WP) at the rate of 0.75 g/kg seed to control insect pests and with fungicide (Raxil, Pencycuron 7.5% WP) at a rate of 1.25 g/kg of seed to control soil-borne diseases. Sowing was done manually at a rate of 120 kg/ha during the last week of November in each of the three seasons. Phosphorus was applied as triple superphosphate by furrow placement before sowing at a rate of 43 kg/ha of P_2_O_5_. In each season, the genotypes were evaluated under two nitrogen treatments: optimum (HN) or deficient (LN). to the HN plots, N fertilizer in the form of urea was split applied at the three-leaf stage (second irrigation) and the tillering stage (fourth irrigation) at 86 kg N/ha. No nitrogen fertilizer was applied at the LN plots, and the plants were left to grow based on the available nitrogen in the soil. In the first (2018/19), second (2019/20), and third (2020/21) seasons, the HS-LN treatments were designated as E1, E3, and E5, respectively, while the HS-HN treatments were designated as E2, E4, and E6. Irrigation was performed every 10-12 days. The field plots were kept weed-free during the growing season.

The MSD lines were phenotyped for 18 phenological, morphological, and physiological traits, including days to heading (DH), days to maturity (DM), grain filling duration (GFD), plant height (PH), spike/m^2^ (SPM), and normalized difference vegetative index (NDVI). The NDVI was assessed during the grain-filling phase using a handheld optical sensor (GreenSeeker, Trimble Inc., Sunnyvale, CA, USA) positioned 50 cm above the center of each plot to measure the plant canopy. Grains per spike (GPS) and thousand kernel weight (TKW) were measured from a subsample of 10 spikes taken from the inner rows. At maturity, the whole plot was harvested from the ground level, and biomass (BIO, g/m^2^), grain yield (GY, g/m^2^), and straw yield (SY, g/m^2^) were determined and harvest index (HI, %), grain numbers/m^2^ (GN) were calculated. Vegetative growth rate (VGR), biomass growth rate (BGR), and grain growth rate (GGR) were calculated by dividing the SY, BIO, and GY by DH, DM, and GFD, respectively. Oven-dried grain samples were milled into fine powder using a grinding mill. We used a CN Corder (Model MT-700; Yanaco, Inc., Kyoto, Japan) to quantify the nitrogen concentration of approximately 50 mg of whole meal drawn from a larger sample. Grain N uptake (GNUp, g/m^2^) was calculated as grain nitrogen content multiplied by grain yield (GNC × GY).

The following indices were calculated for selected traits to compare the genotype performance under combined heat-nitrogen stress (HS-LN) with that under heat stress-high nitrogen (HS-HN).

The relative performance (RP%):


$$\frac{\text{Phenotypic value at HS}-\text{LN}\times 100}{\text{Phenotypic value at HS}-\text{HN}}$$

The Stress tolerance index (STI) by (Fernandez, 1992):


$$\frac{\left(Y_{s})(Y_{p}\right)}{{\left(Y_{p}\right)}^{2}}$$

Where $Y_{s}$ and $Y_{p}$ are the mean phenotypic values of genotypes under stress and non-stress conditions, respectively. Genotypes with high RP and STI values were regarded as tolerant to the combined heat-nitrogen stress.

### 2.3 Statistical analysis

Phenotypic data were analyzed using REML (restricted maximum likelihood) in GenStat software (24^th^ edition VSNi Hemel Hempstead, UK, http://www.genstat.co.uk). The best linear unbiased estimates (BLUEs) of each trait for each genotype in each environment were calculated. The genotype (G), environment (E), and their interaction (G × E) were modeled as the fixed term, while blocks nested within replications were modeled as the random term. The trait BLUEs were utilized in GWAS and all other analyses involving phenotypic data. PBTools software was used to estimate broad-sense heritability (*H* ^2^). AMMI (additive main effects and multiplicative interaction) analysis was conducted using GenStat software, 24^th^ edition. Pearson correlation coefficients and principal component analysis (PCA) were calculated using IBM SPSS Statistics 29.0.2.0 and R software version 4.3.3.

### 2.4 Genotyping and genome-wide association analysis

DNA extraction and genotyping were as described in our previous work (Emam et al., 2025). Genomic DNA was isolated from all genotypes, and two genotyping systems were used in this study: Diversity Array Technology (DArT) and genotyping by Random Amplicon Sequencing-Direct (GRAS-Di) markers. The DNA samples were sent to DArT Pty Ltd, Australia, and Eurofins Genomics K.K., Japan, for whole genome scanning using DArTseq technology and GRAS-Di markers, respectively. DArTseq generated 90,942 markers, including silico DArT (presence/absence) and SNP markers, whereas GRAS-Di generated 17,000 presence/absence markers. We excluded markers with unknown chromosome positions, minor allele frequencies (MAF) of less than 0.01, and more than 10% missing data from both marker sets. The remaining 31,362 markers (4,544 SNPs and 26,818 presence/absence markers) from DArTseq and GRAS-Di were used for association analysis. Linkage disequilibrium (LD) analysis was performed as previously reported (Itam et al., 2022).

We conducted a genome-wide association study (GWAS) to detect the association between SNPs and specific traits using the R software package (Genome Association and Prediction Integrated Tool (GAPIT version 3) (Lipka et al., 2012). Two GWAS models were used: Bayesian information and Linkage disequilibrium Iteratively Nested Keyway (BLINK) (Huang et al., 2019) and Fixed and Random Circulating Probability Unification Model (FarmCPU) (Liu et al., 2016). We used two methods to determine the statistical significance threshold in GWAS: the less conservative -log_10_(*P*) ≥ 3.0 and the more conservative Bonferroni correction. Using the -log_10_(*P*) ≥ 3.0 arbitrary threshold, we detected more than 700 MTAs for different traits, which could result in more false positives, but fewer false negatives. Therefore, we limited its use to the most important traits, such as GNUp and its RP (RP-GNUp) and STI (STI-GNUp). Then, to reduce the risk of false positives (Type I errors), we applied the Bonferroni correction. The Bonferroni correction was applied to determine the statistical significance threshold in GWAS. To determine the Bonferroni correction, the alpha level was set at 0.05 and divided by the total number of markers. The marker-trait associations (MTAs) were considered significant at -log10-6, and R² was used to describe the percentage of variation explained (PVE) by the significant MTAs.

For gene annotation, we employed important MTAs and conducted a BLAST search (https://urgi.versailles.inra.fr/blast/) against *T. aestivum* (Chinese Spring cv. IWGSC RefSeq annotation v2.1). We focused on high-confidence genes located within a range of ± 0.5 Mb upstream and downstream of the significant marker’s position.

## 3 Results

### 3.1 Weather conditions during the cropping seasons

The minimum, maximum, and mean temperatures during the three growing seasons (2018/19-2020/21) at the Wad Medani Experimental Station showed seasonal variations at different growth stages of the crops (**Table 1**). The climatic data from sowing to heading were unavailable for the 2020/21 season. The minimum temperatures from sowing to heading and during GFP (grain filling period) were lowest in 2019/20 (7.3 and 9.8°C, respectively) and highest in 2018/19 (11.0 and 15.5°C, respectively), while it was 11.4°Cduring GFP in 2020/21. The maximum temperatures during both growth stages followed almost the same trend, with the 2018/19 season recording the highest temperatures.

**Table 1:** Duration from sowing to heading, grain filling period, minimum, maximum, and mean temperatures, number of days with temperature ≥ 35°C, number of days with temperature ≤ 15°C, and growing degree days (GDD) at two growth stages of experiments grown at Wad Medani, Sudan during 2018/19-2020/21.

Variables	Growth stage	Season		
		2018/19	2019/20	2020/21
Phenology	Sowing to heading (days)	57	61	58
	Grain filling period (GFP, days)	28	35	35
Minimum temperature, °C	Sowing to heading	11.0	7.3	NA
	GFP	15.5	9.8	11.4
Number of days with temperature ≤ 15°C	Sowing to heading	15	32	NA
	GFP	0	22	12
Maximum temperature, °C	Sowing to heading	39.5	38.5	NA
	GFP	41.0	38.5	37.3
Number of days with temperature ≥ 35°C	Sowing to heading	29	20	NA
	GFP	17	13	3
Mean temperature, °C	Sowing to heading	25.6	23.7	NA
	GFP	27.3	24.2	23.8
GDD (BT=10°C)	Sowing to heading	890.8	835.3	NA
	GFP	485.0	496.9	481.3

NA, data not available.

The number of days with temperatures ≤ 15°C and ≥ 35°C during both growth stages also varied between seasons. The number of days with temperatures ≤ 15°Cfrom sowing to heading in 2019/20 was more than double that in 2018/19. No temperatures below 15°C were recorded during the GFP in 2018/19, but 22 and 12 days were recorded in 2019/20 and 2020/21, respectively. The number of days with high temperatures (≥ 35°C) during both growth stages followed a similar trend, with the highest number recorded in 2018/19. During the GFP, the days with temperatures ≥ 35°C decreased from 17 days in 2018/19, 13 days in 2019/20, to only three days in 2020/21 (**Table 1**).

The growing degree days (GDD) at a base temperature of 10°C varied between seasons. The GDD from sowing to heading decreased from 890.8 in 2018/19 to 835.3 in 2019/20, with no data available for 2020/21. However, during GFP, GDD remained relatively stable across the three seasons, varying from 481.3 in 2020/21, 485.0 in 2018/19, to 496.9 in 2019/20, probably influenced by the duration of the GFP (**Table 1**). These climatic variations highlighted the inter-seasonal variability of heat stress intensity during reproductive development in the heat-prone and dry environments of central Sudan (Gezira State), which was reflected in the crop performance. The responses of the MSD line to these seasonal variations, coupled with combined heat stress-low nitrogen (HS-LN) and heat stress-high nitrogen (HS-HN) treatments, are presented hereafter.

### 3.2 Phenotypic variation and heritability

The REML analysis revealed that environment and genotype effects exerted highly significant (P < 0.001) effects on nearly all studied traits. An exception was observed for GGR, in which the environmental effect was not significant. In addition, genotype-by-environment interactions were significant for all traits, except NDVI and GNC (**Table 2**).

**Table 2:** Analysis of variance, range, mean, and heritability (*H* ^2^) of 18 traits studied in multiple synthetic derivatives population evaluated at Wad Medani under two different conditions: Heat stress-high nitrogen (HS-HN) and combined heat and low nitrogen stress (HS-LN) in three growing seasons, 2018/19, 2019/20, and 2020/21.

Variables	DH	DM	GFD	PH	SPM	GPS	BIO	HI	GY	GN	SY	BGR	VGR	GGR	NDVI	TKW	GNC	GNUp
HS-LN																		
Range	50.3 66.9	79.5 - 97.8	28.1 - 36.9	56.6 - 91.0	273 - 517	18.5 - 44.0	400 - 1018	23.4 - 43.5	131 - 385	3260 - 11713	257.0 - 707.8	50.4 - 113.4	49.7 - 123.0	42.1 - 128.3	0.3 - 0.5	32.6 - 47.8	1.7 - 2.3	2.5 - 9.0
Mean	57.3	88.8	31.4	73.9	379	31.6	772	35.2	268	7066	504	87.2	88.1	86.5	0.36	39.2	2.01	5.1
Norin 61	53.3	82.2	29.0	69.3	347	33.9	745	42.4	315	9264	430	90.1	80.6	109.0	0.38	36.8	1.93	5.6
HS-HN																		
Range	52.8 - 68.1	83.8 - 102.2	29.0 - 39.4	63.3 - 97.6	293 - 564	20.5 - 43.0	583 - 1380	18.3 - 39.9	137 - 442	3897 - 13259	350 - 1021	69.2 - 143.1	64.0 - 173.9	47.1 - 137.8	0.3 - 0.5	29.2 - 44.2	2.2 - 2.8	3.5 - 11.1
Mean	59.4	93.4	33.9	79.1	424	32.2	980	32.0	311	8633	671	104.99	113.01	92.68	0.36	36.91	2.47	7.1
Norin 61	55.2	89.5	34.3	75.9	360	30.3	986	37.9	362	9704	624	109.9	113.6	106.5	0.33	38.3	2.21	7.7
Source of variation																		
Environment	<0.001	<0.001	<0.001	<0.001	<0.001	<0.001	<0.001	<0.001	<0.001	<0.001	<0.001	<0.001	<0.001	0.076	<0.001	<0.001	<0.001	<0.001
Genotype	<0.001	<0.001	<0.001	<0.001	<0.001	<0.001	<0.001	<0.001	<0.001	<0.001	<0.001	<0.001	<0.001	<0.001	<0.001	<0.001	<0.001	<0.001
G x E	<0.001	<0.001	<0.001	<0.001	0.044	<0.001	<0.001	<0.001	<0.001	<0.001	<0.001	<0.001	<0.001	<0.001	0.294	<0.001	0.496	<0.001
% Reduction	3.50	4.94	7.44	6.58	10.62	1.97	21.24	-10.05	13.28	18.15	24.89	16.92	22.01	6.71	-0.47	-6.15	18.55	28.20
*H* ^2^	0.92	0.88	0.78	0.95	0.78	0.88	0.74	0.90	0.86	0.88	0.73	0.66	0.63	0.86	0.75	0.88	0.85	0.72

DH, Days to Heading; DM, Days to maturity; GFD, Grain filling duration; PH, Plant height; SPM, Spike/m^2^; GPS, Grains/spike; BIO, Biomass g/m^2^; HI, Harvest index; GY, Grain yield g/m^2^; GN, Grain numbers/m^2^; SY, Straw yield g/m^2^; BGR, Biomass growth rate; VGR, Vegetative growth rate; GGR, Grain growth rate; NDVI, Normalized differences vegetative index; TKW, 1000 Kernel weight; GNC, Grain nitrogen content%; GNUp, Nitrogen yield g/m^2^.

**Figure 1:**
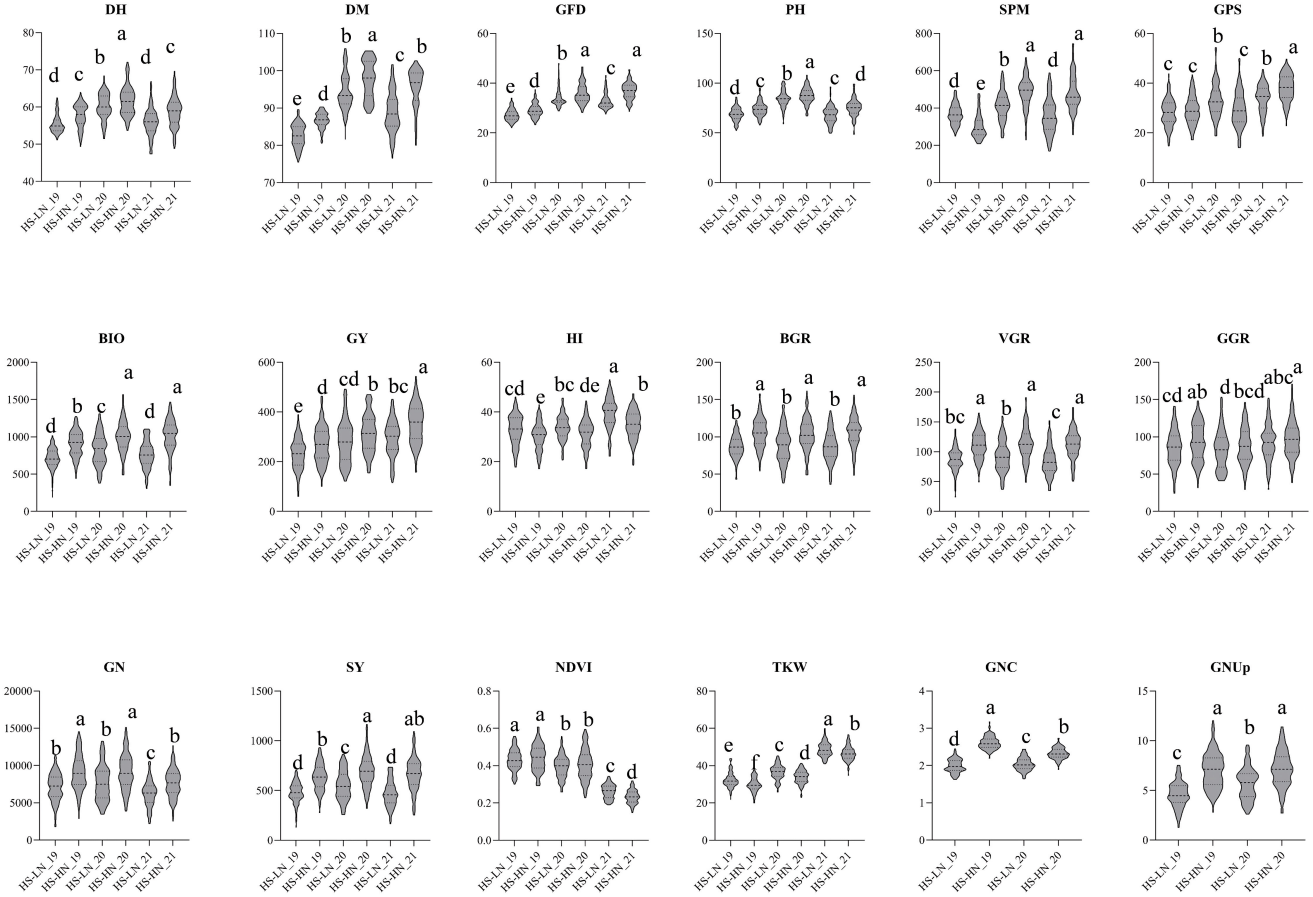
Violin plots for the 18 traits studied in multiple synthetic derivatives population evaluated at Wad Medani under two different conditions: Heat stress-high nitrogen (HS-HN) and Combined heat and low nitrogen stress (HS-LN) in three growing seasons, 2018/19, 2019/20, and 2020/21. Different letters indicate significant differences at *P*<0.05.

Under HS-HN conditions, the MSD lines showed a GY range of 137-442 g/m^2^ with a mean of 311 g/m^2^, whereas N61 produced a GY of 362 g/m^2^. Interestingly, although the biomass of N61 (986 g/m^2^) was similar to the MSD mean, the MSD lines showed a broader range of 583.1-1380 g/m^2^ (**Table 2**; **Figure 1**). This increase in biomass was accompanied by a lower harvest index (HI) in the MSD lines, 32.0% compared to 37.9% in N61, suggesting a trade-off in the partitioning efficiency. Other traits, such as plant height (PH) and grain nitrogen content (GNC), also varied widely among the MSD lines (63.3-97.6 cm and 2.22-2.84%, respectively), with N61 typically aligning near the MSD mean, reflecting its stability under stress (**Table 2**; **Figure 1**).

The mean GY (g/m^2^) decreased from 311 g/m^2^ under HS-HN to 269 g/m^2^ under combined stresses (HS-LN). MSD lines showed greater variation, ranging from 131-385 g/m^2^, with MSD181, MSD026, and MSD485 obtaining significantly higher GY. Similarly, most traits were adversely affected under HS-LN, with spike per meter square (SPM), grain number per square meter (GN), and plant height (PH) being among the most negatively affected traits (**Table 2**; **Figure 1**). In contrast, traits such as the thousand kernel weight (TKW), harvest index (HI), and NDVI increased under HS-LN. (**Table 2**; **Figure 1**). Among physiological traits, the mean GNUp, decreased from 7.1 g/m^2^ under HS-HN to 5.1 g/m^2^ under HS-LN. Similarly, straw yield (SY, g/m^2^), grain nitrogen content (GNC), and biomass growth rate (BGR) were negatively affected (**Table 2**; **Figure 1**).

The MSD lines showed a wide range of variation in GNUp under both conditions, with values ranging from 3.5 to 11.1 g/m^2^ at HS-HN and 2.5 to 9.0 g/m^2^ at HS-LN. Meanwhile, the recurrent parent N61 scored 7.1 and 5.6 g/m^2^ under HS-HN and HS-LN, respectively (**Table 2**; **Figure 1**).

The broad sense heritability (*H*²) of the traits studied ranged from 0.63 to 0.95 (**Table 2**). High *H*² values (≥ 0.85) were observed for DH, DM, PH, HI, GPS, GY, GN, TKW, GGR, and GNC, indicating strong genetic control of these traits. The *H*² of the GFD, SPM, NDVI, BIO, SY, and GNUp ranged from 0.72 to 0.78. The *H*² of BGR (*H*² =0.66) and VGR (*H*² = 0.63) were intermediate and more influenced by environmental factors (**Table 2**).

### 3.3 Identification of stress resilient lines

To assess the stress resilience of the MSD lines, we calculated the stress tolerance index (STI) and relative performance (RP%) of selected traits based on the performance under combined heat-nitrogen stress (HS-LN) compared to that under heat stress-high nitrogen (HS-HN) (**Supplementary Table 1**). Based on the grain yield STI (STI-GY), more than 20 MSD lines and the Sudanese cultivar Imam were more resilient than the MSD parent N61 (**Supplementary Table 1**). Among these resilient lines, some exhibited higher STI values for GN (STI-GN), GNUp (STI-GNUp), SPM (STI-SPM), and GPS (STI-GPS); specifically, MSD024 demonstrated resilience based on indices of multiple traits. MSD lines MSD024, MSD485, MSD490, MSD181, MSD317, MSD413, and MSD026 emerged as top performers, showing both favorable yield-related traits and relatively stable GNUp and GNC under combined stresses (**Supplementary Table 1**).

According to AMMI analysis for GY, genotype, environment, and their interaction showed significant effects, explaining 35.2, 18.8, and 46.0%, respectively, of the phenotypic variation (**Supplementary Table 2**). Distinct genotypic responses were found across the six environments under heat stress conditions (HS-HN) and combined heat-nitrogen stress (HS-LN). The MSD lines MSD053, MSD450, MSD017, and MSD361 were in the top 10% under HS-HN (responsive), while they were not in the top 25 lines under HS-LN (**Table 3**). On the other hand, MSD192, MSD163, MSD383, and MSD254 were in the top 10% under HS-LN (efficient), while they were not in the top 25 lines under HS-HN. The MSD line MSD026 ranked highest under HS-LN, HS-HN, and the overall mean. Similarly, MSD181 and MSD485 were among the top five ranking lines under the two conditions and the overall mean (**Table 3**).

**Table 3:** The top 20 lines, according to AMMI estimates for grain yield, under HS-LN, HS-HN, and the overall mean.

Genotype	E1 (HS-LN)	E2 (HS-HN)	E3 (HS-LN)	E4 (HS-HN)	E5 (HS-LN)	E6 (HS-HN)	HS-LN Mean (Rank)	HS-HN Mean (Rank)	Overall mean	ASV	GSI	Overall mean Rank
MSD017	295.1 (19)	415.6 (3)	322.2 (46)	445.1 (6)	303.8 (77)	331.7 (93)	307.0 (32)	397.5 (6)	352.3 (19)	4.73	128	33.4
MSD024	314.9 (9)	306.8 (46)	453.6 (6)	381.3 (26)	372.5 (16)	423.7 (21)	380.3 (2)	370.6 (18)	375.5 (4)	3.51	83	16.4
MSD026	323.7 (7)	362.5 (13)	346.2 (33)	458.7 (5)	475.6 (1)	514.2 (1)	381.8 (1)	445.1 (1)	413.5 (1)	3.39	77	7.0
MSD041	285.9 (23)	284.0 (64)	348.4 (30)	311.8 (73)	367.1 (17)	436.6 (14)	333.8 (20)	344.1 (42)	339.0 (25)	2.05	61	34.2
MSD044	229.2 (75)	218.8 (113)	459.8 (5)	319.2 (68)	224.4 (127)	260.4 (133)	304.5 (35)	266.1 (120)	285.3 (86)	8.74	231	84.7
MSD053	331.0 (4)	477.9 (1)	270.8 (79)	410.2 (16)	306.3 (73)	360.9 (72)	302.7 (39)	416.3 (3)	359.5 (14)	6.01	143	33.4
MSD112	263.4 (41)	311.4 (42)	285.6 (71)	386.0 (23)	386.3 (11)	427.8 (19)	311.8 (27)	375.1 (15)	343.4 (22)	2.24	64	30.1
MSD117	311.9 (12)	373.5 (11)	452.2 (7)	437.4 (7)	295.8 (88)	324.7 (98)	353.3 (12)	378.5 (14)	365.9 (11)	7.10	148	28.9
MSD135	313.1 (11)	414.8 (5)	432.6 (8)	429.7 (13)	233.5 (119)	263.9 (130)	326.4 (21)	369.5 (20)	347.9 (20)	9.51	168	38.6
MSD145	220.2 (87)	320.8 (34)	323.6 (44)	496.2 (1)	315.9 (68)	301.5 (115)	286.6 (54)	372.8 (16)	329.7 (32)	4.65	140	50.1
MSD163	252.3 (55)	230.5 (103)	478.4 (2)	436.7 (10)	366.5 (19)	376.3 (56)	365.7 (8)	347.8 (37)	356.8 (16)	7.41	157	34.0
MSD181	333.3 (3)	407.6 (6)	423.6 (9)	483.3 (2)	366.7 (18)	392.0 (47)	374.5 (4)	427.6 (2)	401.1 (2)	4.19	97	10.3
MSD189	292.6 (21)	308.8 (43)	298.4 (59)	360.2 (42)	436.7 (2)	497.9 (2)	342.6 (15)	389.0 (10)	365.8 (12)	4.95	127	22.9
MSD192	309.6 (13)	304.7 (52)	471.2 (3)	384.8 (24)	347.6 (34)	393.9 (41)	376.1 (3)	361.1 (26)	368.6 (8)	4.83	120	22.7
MSD249	313.8 (10)	299.8 (57)	383.1 (16)	337.8 (49)	409.1 (6)	479.4 (5)	368.7 (7)	372.3 (17)	370.5 (7)	2.84	62	19.3
MSD254	324.7 (6)	320.3 (35)	388.8 (13)	292.2 (84)	351.3 (30)	437.9 (13)	354.9 (10)	350.1 (32)	352.5 (18)	0.59	21	26.8
MSD257	269.6 (29)	228.7 (104)	359.1 (25)	281.1 (92)	383.0 (13)	457.0 (9)	337.2 (18)	322.3 (68)	329.8 (31)	4.15	124	43.2
MSD275	264.2 (37)	265.9 (79)	373.4 (17)	362.7 (39)	364.4 (20)	408.2 (28)	334.0 (19)	345.6 (39)	339.8 (24)	2.62	75	33.6
MSD317	306.9 (14)	337.6 (27)	464.1 (4)	435.5 (11)	335.1 (48)	363.6 (69)	368.7 (6)	378.9 (13)	373.8 (5)	5.86	132	21.9
MSD361	278.5 (27)	337.8 (26)	203.0 (116)	326.6 (61)	402.3 (7)	473.6 (6)	294.6 (49)	379.3 (12)	337.0 (26)	6.71	161	36.7
MSD378	218.6 (92)	261.3 (83)	250.1 (91)	414.7 (14)	414.7 (5)	434.4 (15)	294.5 (50)	370.1 (19)	332.3 (30)	4.08	121	44.3
MSD383	361.9 (1)	415.4 (4)	480.1 (1)	334.9 (52)	232.2 (120)	308.0 (111)	358.1 (9)	352.8 (30)	355.4 (17)	8.46	161	38.3
MSD401	252.5 (54)	280.3 (70)	384.6 (15)	466.2 (4)	395.1 (8)	401.4 (35)	344.1 (14)	382.6 (11)	363.4 (13)	3.79	99	24.9
MSD413	256.9 (50)	303.6 (54)	372.3 (18)	482.7 (3)	392.5 (9)	395.5 (40)	340.6 (17)	393.9 (7)	367.3 (10)	3.20	81	23.1
MSD426	257.1 (48)	222.7 (109)	385.9 (14)	332.0 (55)	382.6 (14)	434.1 (16)	341.9 (16)	329.6 (58)	335.7 (27)	4.17	121	39.7
MSD427	302.3 (17)	315.0 (39)	185.9 (127)	137.4 (148)	343.5 (40)	483.8 (3)	277.2 (66)	312.1 (79)	294.7 (74)	9.32	221	65.9
MSD450	325.7 (5)	428.8 (2)	297.1 (61)	377.3 (28)	324.6 (59)	387.4 (50)	315.8 (26)	397.8 (5)	356.8 (15)	4.00	105	27.9
MSD485	320.6 (8)	339.9 (25)	371.0 (19)	399.3 (19)	425.8 (4)	479.5 (4)	372.5 (5)	406.2 (4)	389.4 (3)	2.06	41	10.1
MSD490	262.0 (45)	276.8 (75)	342.6 (34)	430.9 (12)	435.3 (3)	460.4 (8)	346.6 (13)	389.4 (9)	368.0 (9)	3.63	91	23.1
Norin 61	264.8 (35)	284.2 (63)	311.2 (53)	358.3 (44)	388.3 (10)	438.1 (12)	321.4 (24)	360.2 (27)	340.8 (23)	2.69	76	32.3
Imam	337.6 (2)	403.6 (7)	406.6 (10)	395.5 (20)	316.5 (67)	371.2 (60)	353.6 (11)	390.1 (8)	371.8 (6)	4.28	104	21.2

The top five lines in each environment were also retained. The AMMI stability value (ASV) and genotype selection index (GSI) for grain yield are also shown. The numbers in parentheses are the overall rank of each genotype at each environment, HS-LN, HS-HN, and the overall mean. The overall mean rank is the average of all ranks in all environments.

### 3.4 Trait associations

**Figure 2:**
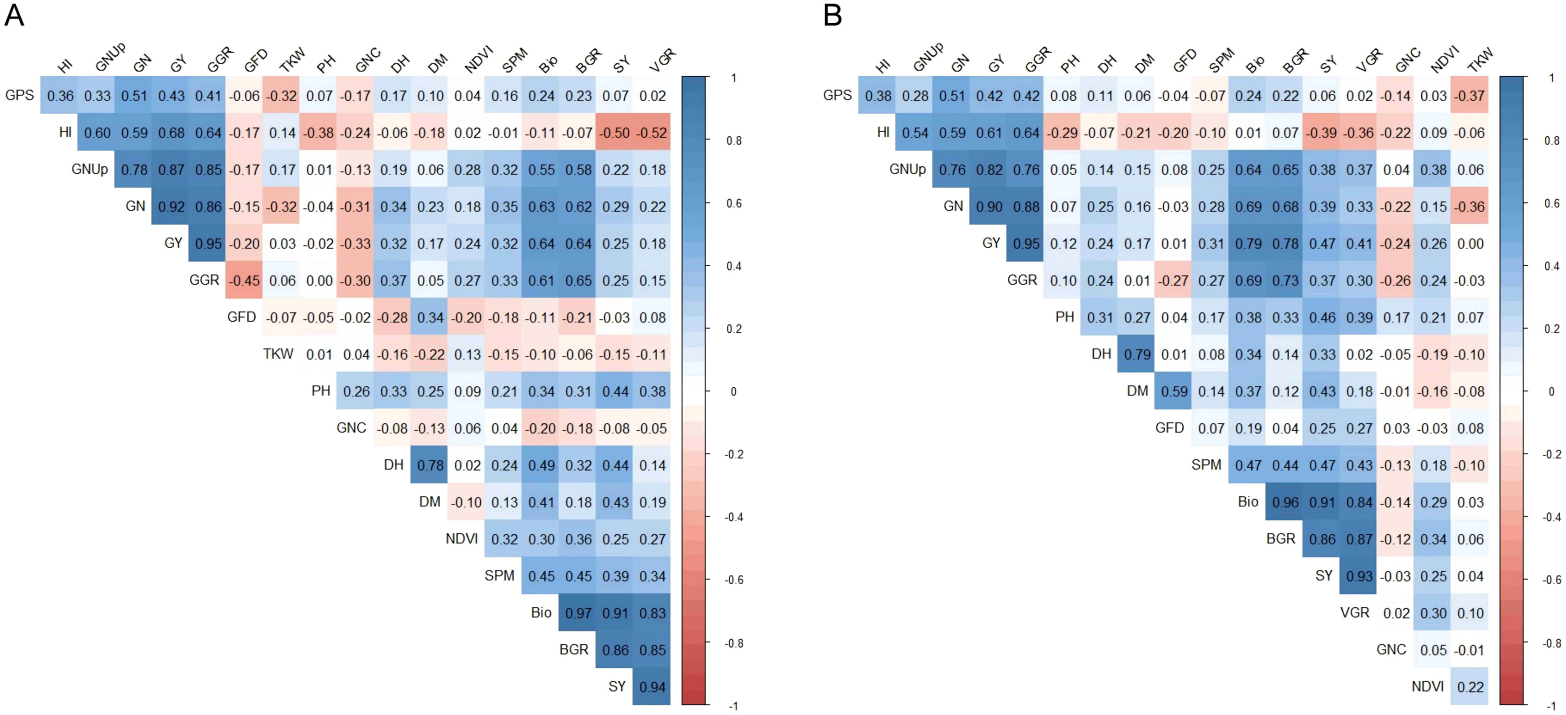
Pearson correlation among of 18 traits measured based on the combined mean of 148 lines evaluated at Wad Medani under two different conditions: **(A)** heat stress-high nitrogen (HS-HN) and **(B)** Combined heat and low nitrogen stress (HS-LN) in three growing seasons, 2018/19, 2019/20, and 2020/21.

Pearson’s correlation coefficients revealed strong positive relationships among yield-related traits (SPM, GPS, GN, and GY), biomass-associated traits (BIO and BGR), and HI under both HS-HN and HS-LN (**Supplementary Table 3**; **Figures 2A, B**). Nitrogen-related traits, particularly GNUp, were positively correlated with grain yield in both treatments, whereas GNC showed an inverse relationship with yield and the other traits. TKW was generally negatively correlated with most traits studied (**Supplementary Table 3**; **Figures 2A, B**). Under HS-HN, notably, GY was highly correlated with grain number per m² (GN) and grain growth rate (GGR), with coefficients greater than 0.90 (**Supplementary Table 3**; **Figure 2A**). GY also showed moderate positive correlations with total biomass g/m^2^ (BIO) and harvest index (HI). Under HS-LN, a similar overall pattern of trait correlation was observed, with some shifts in magnitude (**Supplementary Table 3**; **Figure 2B**). GY remained the most strongly associated with GN and GGR. Interestingly, the correlation between GY and BIO became stronger under low N stress with 0.79 at HS-LN compared to 0.64 at HS-HN. GY was also strongly correlated with BGR and GNUp.

**Figure 3:**
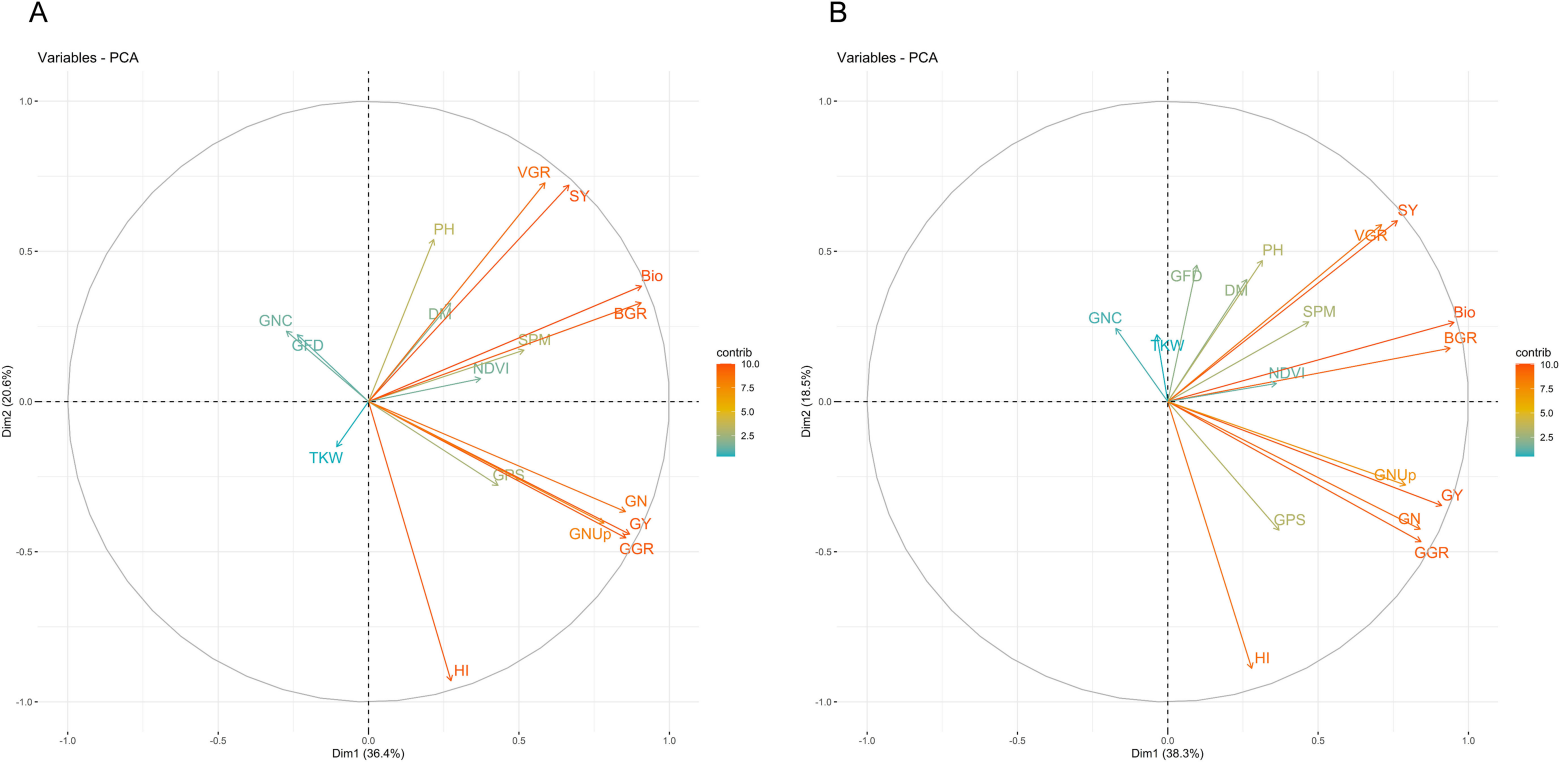
Principal component analysis (PCA) of 18 traits measured based on the combined mean of 148 lines grown under two different conditions: **(A)** heat stress-high nitrogen (HS-HN) and **(B)** combined heat and low nitrogen stress (HS-LN) in three growing seasons, 2018/19, 2019/20, and 2020/21.

Principal component analysis (PCA) further emphasized these trait associations. Under HS-HN, the first principal component (PC1) explained 35.6% of the variation, primarily driven by GY, HI, and BIO, whereas PC2 accounted for 19.7%, influenced by TKW, grain-filling duration (GFD), and GNC (**Figure 3A**). The GY, GNUp, GPS, and GN clustered closely, indicating strong positive correlations. BIO, BGR, and SPM formed tight clusters. TKW, GFD and GNC stood apart and were negatively correlated with other key traits. Under HS-LN, PC1 explained 36.7% of the variation and was again mainly associated with GY, HI, and BIO, whereas PC2 explained 17.7% and was influenced by TKW and GNC (**Figure 3B**). The separation of TKW, GFD and GNC from the yield and biomass components remained consistent in HS-HN and HS-LN, suggesting the strong influence of heat stress.

### 3.5 Genome-wide association analysis

A GWAS was conducted on 18 phenotypic traits using 31,362 high-quality markers to uncover the genetic loci involved in wheat tolerance to heat stress under sufficient and deficient nitrogen conditions (**Table 4**; **Supplementary Figure 1**). This panel, described earlier by (Itam et al., 2022) and (Emam et al., 2025), shows an LD decay of 131 Mb in the A and B subgenomes and a markedly lower LD decay of 1.1 Mb in the D subgenome. Significant marker-trait associations (MTAs) were identified using the BLINK and FarmCPU.

**Table 4:** Significant marker trait associations at Bonferroni corrected p-values for traits under study evaluated at Wad Medani under two different conditions: Heat stress-high nitrogen (HS-HN) and combined heat and low nitrogen stress (HS-LN) in three growing seasons, 2018/19, 2019/20, and 2020/21.

SN	Environment	Trait	SNP	Chr	Models	Pleiotropic/Stable	Models	Postion	*P*.value	PVE (%)
1	HS-LN	PH	AMP0042645	4D	BLINK		BLINK/FarmCPU	25,795,214	0.000000000	36.03
2	HS-LN	PH	AMP0059473	6D	BLINK		BLINK/FarmCPU	117,443,593	0.000000509	13.81
3	HS-LN	GGR	rs#987242	5A	BLINK	Stable	BLINK/FarmCPU	572,991,306	0.000000001	36.84
4	HS-HN	DH	AMP0075964	4B	BLINK			639,800,091	0.000000017	13.12
5	HS-HN	DH	AMP0011184	7B	BLINK			12,755,705	0.000000001	15.23
6	HS-HN	DH	AMP0030874	7D	BLINK			403,624,789	0.000000000	13.72
7	HS-HN	PH/GNC	AMP0015831	4D	BLINK	Pleiotropic	BLINK/FarmCPU	18,724,155	0.000000000	56.46
8	HS-HN	GPS	AMP0068214	1D	BLINK		BLINK/FarmCPU	235,563,193	0.000000002	68.16
9	HS-HN	HI	AMP0011306	2A	BLINK			37,514,981	0.000000426	12.57
10	HS-HN	HI	AMP0075052	2D	BLINK			22,954,672	0.000000005	28.34
11	HS-HN	GY	rs#987242	5A	BLINK	Pleiotropic		572,991,306	0.000000002	40.39
12	HS-HN	GGR	AMP0017606	4D	BLINK			13,276,371	0.000000270	9.84
13	HS-HN	GGR	rs#987242	5A	BLINK	Stable	BLINK/FarmCPU	572,991,306	0.000000000	20.91
14	HS-HN	GGR	rs#1090452	6D	BLINK			119,244,728	0.000000270	18.66
15	HS-HN	GNUp	AMP0007457	5A	BLINK		BLINK/FarmCPU	576,110,337	0.000000006	40.39
16	STI/RP	RP-GNUp	rs#1071033	3D	BLINK		BLINK/FarmCPU	100,931,281	0.000000000	87.62
17	HS-HN	PH	AMP0066047	4D	FarmCPU			67,102,344	0.000000304	21.45
18	HS-HN	PH	AMP0030888	6B	FarmCPU			189,584,852	0.000000000	27.42
19	HS-HN	PH	AMP0013116	6D	FarmCPU			356,748,918	0.000000810	7.71
20	HS-HN	GPS	rs#3941273	1D	FarmCPU			362,470,929	0.000000253	3.50
21	HS-HN	GPS	rs#979901	1D	FarmCPU			431,160,165	0.000000196	15.03
22	HS-HN	GPS	rs#5580164	6B	FarmCPU			474,860,950	0.000000024	5.32
23	HS-HN	GNC	rs#1102535	4D	FarmCPU			12,166,393	0.000000779	12.69
24	HS-HN	GNUp	rs#1165624	3D	FarmCPU			18,053,868	0.000000025	3.67
25	HS-HN	GNUp	rs#1115935	5B	FarmCPU			240,950,284	0.000000579	3.17
26	STI/RP	RP-GNUp	AMP0090426	2A	FarmCPU			93,057,859	0.000000005	2.06
27	STI/RP	RP-GNUp	AMP0070654	4D	FarmCPU			383,936,983	0.000000000	27.25
28	STI/RP	RP-GNUp	AMP0033222	5A	FarmCPU			685,720,022	0.000000776	0.18

STI, Stress tolerance index; RP, Relative performance%; DH, Days to Heading; PH, Plant height; GPS, Grains/spike; HI, Harvest index; GY, Grain yield g/m^2^; GGR, Grain growth rate; TKW, 1000 Kernel weight; GNC, Grain nitrogen content%; GNUp, Nitrogen yield g/m^2^; SNP, Single nucleotide polymorphism.

Due to the large number of MTAs detected, we selectively applied the -log_10_(*P*) ≥ 3.0 threshold to GNUp, RP-GNUp, and STI-GNUp. A total of 140 MTAs were identified: 69 for GNUp under both HS-HN and HS-LN conditions, 43 for RP-GNUp, and 28 for STI-GNUp (**Supplementary Table 4**). At the D subgenome, 80 MTAs were detected across all seven chromosomes (1D-7D).

A total of 34 highly significant MTAs were identified and were distributed across 12 chromosomes (1D, 2A, 2D, 3D, 4 B, 4D, 5A, 5 B, 6 B, 6D, 7 B, and 7D) (**Table 4**). Twenty-four MTAs were detected under HS-HN, five under HS-LN, and five were associated with stress tolerance indices (**Table 4**). The PH had the highest number of MTAs (8), followed by GPS (5), and RP-GNUp (5). The identified MTAs were also associated with other important agronomic traits such as DH, GGR, HI, GNC, and GNUp. Only a single MTA was associated with GY. In terms of genomic distribution, most MTAs were found in the D subgenome (61.8%), followed by subgenomes A (23.5%), and B (14.5%). Two pleiotropic or stable MTAs were detected: one linked to GGR under HS-LN and HS-HN, and GNUp under HS-HN (rs#987242), and another associated with GNC and PH under HS-HN (AMP0015831) (**Table 4**). Five MTAs (AMP0042645, AMP0059473, AMP0068214, AMP0007457, and rs#1071033) were identified using both GWAS models, indicating their robustness in different analyses.

Thirteen of the 34 Bonferroni-significant MTAs were mapped to the biparental A/B subgenomes, with eight mapped to the A subgenome and five to the B subgenome. These MTAs were segregated between the durum donor, Langdon, and the recurrent parent, Norin 61. Their effects were mostly neutral or modestly negative. Notably, the Langdon allele at AMP0075964-4B delayed heading time, and the alleles AMP0011306-2A and AMP0007457-5A reduced the harvest index and GNUp. The allele AMP0033222-5A provided the only favorable signal, increasing RP-GNUp (**Supplementary Figure 2**).

### 3.6 Gene annotation and allele contribution

**Figure 4:**
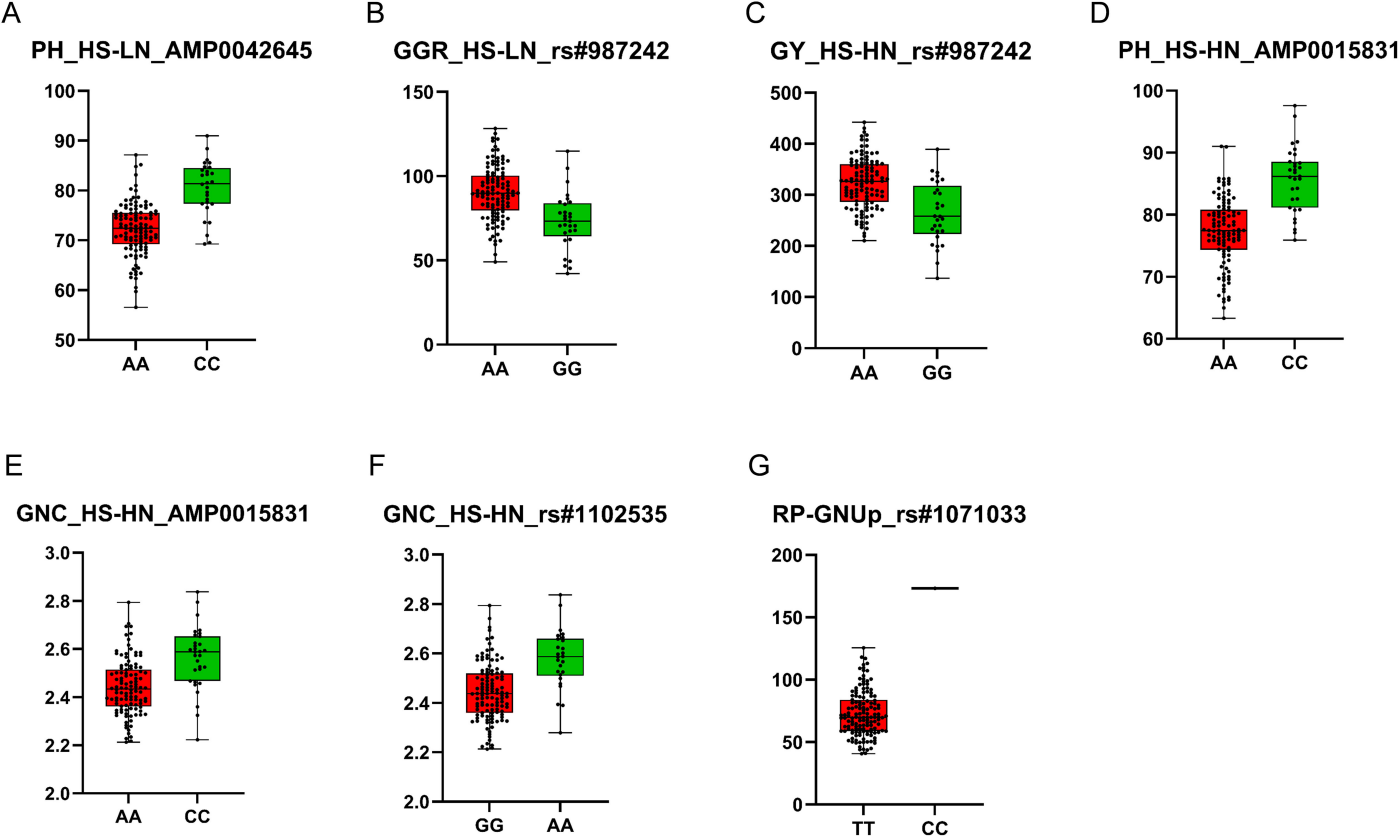
Allelic differences in significant and staple/pleiotropic MTAs, including AMP0042645 **(A)**, rs#987242 **(B, C)**, AMP0015831 **(D, E)**, rs#1102535 **(F)**, and rs#1071033 **(G)**. The lines with the recurrent parent Norin 61 alleles are marked in red, while those containing *Ae. tauschii* are shown in green.

The positions of statistically significant MTAs were explored by subjecting them to a BLAST search to annotate genomic regions and identify nearby potential candidate genes associated with stress responses or key agronomic processes in wheat. A total of 178 candidate genes were associated with significant MTAs at both HS-HN and HS-LN (**Supplementary Table 4**). On chromosome 4D, MTA for PH (AMP0042645) with PVE of 36.0% was found to be related to the candidate gene *TraesCS4D03G0088500*. This candidate gene encodes the mitogen-activated protein kinase (MAPK), which mediates signal transduction under multiple stress conditions, including heat stress and nitrogen deficiency (Wen et al., 2015; Shi et al., 2020; Sultana et al., 2020; Kumar et al., 2021). Lines harboring the A allele from N61 exhibited reduced PH compared to those carrying the C allele from *Ae. tauschii* (**Figure 4**). Several other candidate genes with diverse regulatory functions have been identified. On chromosome 5A, MTA (rs#987242) for GGR and GY with PVE 36.8 and 40.4%, respectively, was related to the candidate gene *TraesCS5A03G0894100*, which encodes a protein containing methyl-CpG-binding domain and appears to influence gene expression and genome stability, thereby facilitating stress adaptation. Another candidate gene on the same locus on chromosome 5A (*TraesCS5A03G0895300*) encodes a DnaJ protein homolog that interacts with Hsp70, regulates ATPase activity, and aids in the transcriptional modification of protective factors under stress. On chromosome 4D, MTA for PH and GNC was related to the candidate gene *TraesCS4D03G0067100* encodes DELLA protein (RHT-1), which modulates gibberellic acid signaling, affects plant height (PH), and enhances tolerance to lodging and adverse environmental conditions. In the MSD population, *Ae. tauschii* genome contributed beneficial alleles that enhanced both plant height (PH) and grain nitrogen content (GNC) under HS-HN conditions, surpassing genotypes carrying the recurrent parent N61 allele (**Figure 4**). One particularly promising locus on chromosome 3D (rs#1071033) was associated with RP-GNUp with a PVE of 87.6% and was located on the candidate gene *TraesCS3D03G0294800*, which encodes a MADS-box transcription factor. The line with alle derived from the wild progenitor showed a significantly higher value of RP-GNUp than those carrying the allele from the recurrent parent N61 (**Figure 4**).

Out of the 140 GNUp MTAs identified applying -log_10_(*P*) ≥ 3.0, four SNPs, AMP0007457, AMP0071287, AMP0023068, and rs#2252596, were stable across environments. A BLAST search at ± 0.5 Mbp distance upstream and downstream of the marker position revealed 54 candidate genes, including transporters and receptor-like kinases involved in nitrogen remobilization and stress signaling (**Supplementary Table 6**).

## 4 Discussion

Maintaining high crop yields while minimizing environmental risks is challenging in the era of climate change and food insecurity. Enhancing crop NUE is a fundamental strategy to achieve these goals (Costanzo and Bàrberi, 2014; Ying et al., 2017; Ain, 2024). In this study, we dissected the genetic and physiological basis of nitrogen use efficiency (NUE) under combined heat and nitrogen stress in six field environments using 145 MSD lines derived from a diverse set of *Ae. tauschii* accessions. Several MSD lines maintained high yield performance, despite the significant reductions in the GY (14%) and GNUp (28%) under combined HS-LN stress conditions compared to those under HS-HN. The identification of 34 marker-trait associations (MTAs), mainly from the D subgenome of *Ae. tauschii*, and the discovery of novel loci associated with NUE, underlined the potential of the MSD to significantly contribute to the breeding of climate-resilient, resource-efficient wheat cultivars.

Besides GY and GNUp, our results from field experiments demonstrated that the combined nitrogen deficiency and heat stress significantly affected most of the studied traits. Biomass and biomass-related traits such as SY, VGR, and BGR were among the traits most affected by HS-LN compared to HS-HN. The combined stress also reduced GN (18.1%) and SPM (10.6%), and to a lesser extent, GPS (2%). In contrast, HI and TKW increased under the combined stress conditions (HS-LN) by 10.1 and 6.7%, respectively, compared to those under HS-HN. The increase in HI was mainly because the reduction in BIO (21.2%) was higher than that of the GY. Increasing N supply from zero to 86 kg N/ha resulted in a 32.4% increase in biomass and a 3.5% reduction in HI of adapted cultivars to the same environment of this study (Tahir et al., 2020). Similarly, the reduction in GN, SPM, and GPS resulted in fewer grains of higher weight. This suggests an adaptive reallocation of resources to grain filling at the expense of total biomass when nitrogen is scarce; therefore, indirect selection based on the relative performance of genotypes for traits such as TKW may be effective in improving grain yield under heat stress (Sharma et al., 2008). These results indicated that breeding for combined stress tolerance should focus on concurrently improving multiple yield components, rather than optimizing single traits. For example, simultaneously enhancing grain number per square meter (GN) and biomass per square meter (BIO) may be more effective in maintaining yield under stress than targeting either trait alone.

Our results of reduced GY and other traits under combined heat stress and nitrogen deficiency conditions are consistent with previous research that reported reduced yield and yield-related traits (Liu et al., 2013; Xu et al., 2022). Besides the morphological processes, nitrogen limitation likely impairs key physiological processes (e.g., chlorophyll synthesis and biomass accumulation), which in turn exacerbates heat stress damage (Wang et al., 2019, 2021). On the other hand, under combined post-anthesis heat and drought stress, reduced N application effectively mitigated yield reduction and improved N use efficiency in wheat (Ru et al., 2022, 2024a, 2024b). However, Tahir et al. (2020) reported significant genetic gains in NUE under high N supply under continuous heat stress field conditions, but not under low N supply.

Despite the overall reduction in most traits, the MSD lines showed considerable differential response to both HS-HN and HS-LN conditions. Selection based on the top-ranked genotypes in GY in each environment showed that different genotypic responses were observed among the six environments under HS-HN and HS-LN. The MSD lines MSD053, MSD450, MSD017, and MSD361 were in the top 10% under HS-HN, but not in the top 25 lines under HS-LN, representing genotypes more responsive to N supply. Conversely, MSD192, MSD163, MSD383, and MSD254 were in the top 10% under HS-LN, but not in the top 25 lines under HS-HN, representing genotypes more efficiently utilizing available N. The third group of genotypes was represented by MSD026, MSD181, and MSD485, which were among the top five-ranked lines under both conditions and across all environments. This group, which was identified as better adapted to most of the environments based on their GY performance, GSI, and overall rank across all environments, represents readily available resources for climate-resilient wheat breeding.

Many traits showed moderate-to-high broad-sense heritability under combined stress, indicating a strong genetic component of their variation. For instance, the broad-sense heritability ranged from 0.63 for VGR to 0.95 for PH, with that of GY, GNC, and GNUp being 0.86, 0.85, and 0.72, respectively. Consistent with these findings, high to moderate heritability values were reported under various growing conditions and environments (Lopes et al., 2013; Elhadi et al., 2021b; Gorafi et al., 2025). The high heritability values and NUE for GY and other related traits reported here are encouraging for breeders, as they suggest that these traits can be effectively improved through selection to enhance stress resilience. However, as broad-sense heritability is a broader indicator, caution should be taken because of significant environmental effects for most quantitative traits.

To identify stress-resilient genotypes, we examined the stress tolerance indices. Higher stress tolerance index (STI) and relative performance (RP%) values generally indicate better tolerance to heat and low-N conditions (Lamba et al., 2023) and nitrogen deficiency (Shi et al., 2022). In our study, GY, GNC, and GNUp under both HS-LN and HS-HN positively correlated with STI and RP%. Using these criteria, we identified several superior lines. Notably, MSD024, MSD485, MSD490, MSD181, MSD317, MSD413, and MSD026 maintained higher grain yield, GNC, and GNUp than the other lines under combined stress. Coupled with selection made from the top-ranking genotypes and the GSI, these indices reliably identify stable and productive genotypes (Gavuzzi et al., 1997; Lamba et al., 2023). The out-performance of these lines may be attributed to the unique alleles introduced by the *Ae. tauschii* ancestor (Gorafi et al., 2018). These top-performing genotypes provide valuable resources for further genetic and physiological studies aimed at improving heat and N stress tolerance in wheat.

Genome-wide association analysis of the MSD population yielded 34 significant marker–trait associations (MTAs) for eight traits, including plant height, grains per spike, relative performance of Grain N uptake (RP-GNUp), and grain yield. To the best of our knowledge, no studies have explored the genetic associations under simultaneous heat and N stress; therefore, these results provide novel insights. Notably, most MTAs were located in the wheat D subgenome, consistent with the crucial stress-adaptive alleles contributed by *Ae. tauschii* (Elhadi et al., 2021b; Itam et al., 2022). This finding is significant because the D subgenome diversity of wheat has been underutilized in breeding (Gorafi et al., 2018; Kishii, 2019). Harnessing these D subgenome loci from exotic sources could enhance wheat breeding under combined stress conditions.

Several MTAs are particularly noteworthy for their pleiotropic effects and novel contributions. The SNP marker rs#987242 on chromosome 5A was associated with grain yield under HS-HN and with the green ground cover ratio (GGR) under both N regimes. Another locus, marked by SNP AMP0015831 on 4D, affected both plant height and grain N content under HS-HN; lines carrying the *Ae. tauschii*–derived allele at this locus was taller and had higher GNC than those with the recurrent parent (N61) allele. In addition, we identified a previously unreported MTA on chromosome 3D (marker rs#1071033) for RP-GNUp, where the wild progenitor allele conferred a significantly higher RP-GNUp than the N61 allele. Xu et al. (2014) examined a population of 182 F11 RILs under three different nitrogen and phosphorus levels and identified one quantitative trait locus (QTL) for GNUp on chromosome 6D under low N conditions. These stress-responsive loci, particularly those with consistent effects across N conditions, are valuable targets for marker-assisted selection to improve wheat performance in both N-poor and N-rich environments.

Genotype-by-environment interactions play a substantial role in trait expression under stressful conditions. We observed that traits, such as days to heading, grain yield, GNUp, and grain N content, differed significantly between the HS-LN and HS-HN treatments, underscoring the complexity of breeding under combined stress conditions. Therefore, identifying loci that confer robust performance across both N-deficient and N-sufficient environments (such as some of the pleiotropic markers mentioned above) is particularly beneficial. Such stable MTAs can help breeders develop wheat varieties that maintain high productivity under variable nitrogen availability.

Our analysis also highlighted putative candidate genes underlying several MTAs, offering insights into stress adaptation mechanisms. For example, a MAP kinase gene (*TraesCS4D03G0088500*) co-located with a 4D MTA (AMP0042645) for plant height (PH), MAPKs, is known to regulate plant responses to heat stress (Kumar et al., 2021) and nitrogen deficiency (Wen et al., 2015; Shi et al., 2020; Sultana et al., 2020). Similarly, a DnaJ protein homolog (*TraesCS5A03G0895300*) interacts with heat shock proteins (HSPs) and plays a role in stabilizing proteins under heat stress (Kong et al., 2014). We also detected a MTA (AMP0015831) for plant height (PH) and GNC overlapping the gene DELLA protein (RHT-1, *TraesCS4D03G0067100*) at 4D, which influences plant height and biomass partitioning (Cheng et al., 2022). In our panel, lines with moderate (semi-dwarf) stature and greater biomass tend to produce higher grain and N yields. Although shorter plants can reduce lodging, breeders should balance plant height with sufficient biomass and stress tolerance to maximize yield under hot, low-nitrogen conditions.

This study focused primarily on the stringent Bonferroni correction to ensure high-confidence associations. However, applying a less conservative threshold (-log_10_(*P*) ≥ 3.0) revealed additional MTAs, some of which were stable across multiple environments. While these findings require further validation, they may point to useful genetic loci that could complement the high-confidence markers identified through the Bonferroni correction. This is particularly relevant for complex traits, where multiple small-effect loci may contribute to phenotypic variation.

In conclusion, this study demonstrates that MSD wheat lines harbor valuable genetic variation in NUE and yield under combined heat and low N stress. The results of this study have significant applied implications for wheat breeding for climate resilience and input efficiency. For instance, MSD lines such as MSD026, MSD181, MSD024, and MSD485 consistently showed superior performance in both combined stress environments, demonstrating yield stability and improved nitrogen uptake efficiency. They are particularly valuable as donors for introgression into elite breeding germplasm due to their favorable genetic backgrounds enriched with *Ae. tauschii*-derived diversity. Furthermore, the identified pleiotropic MTAs, colocalized with genes involved in nitrogen transport (e.g., NRT1/PTR family) and heat response (e.g., HSP genes) are powerful tools for simultaneously selecting multiple favorable traits through marker-assisted selection strategies. The MTAs and candidate genes identified here need to be validated in diverse germplasm and environments to confirm their effectiveness. Additionally, fine-mapping these MTAs and developing near-isogenic lines are important to pinpoint causal alleles and facilitate their deployment in breeding programs. Incorporating elite lines and associated markers into breeding programs can accelerate the development of wheat cultivars for resource-constrained and climate-vulnerable environments.

## Data Availability

The datasets presented in this study can be found in online repositories. The names of the repository/repositories and accession number(s) can be found in the article/**Supplementary Material**.

## Supplementary material

Supplementary Figure 1 GWAS of agronomic, physiological, and NUE-related traits of wheat multiple synthetic derivative (MSD) under heat stress-high N (HS-HN) and heat stress-low N (HS-LN) conditions using Manhattan plot and Q鵬 plot.

Supplementary Figure 2 Allelic effect of durum (Langdon) allele. The lines with the durum (Langdon) allele are marked in red, whereas those containing Ae. tauschii are indicated in green.
