# Supplementary figures and images for: Genetic variation and genome-wide association analysis of nitrogen use efficiency-related traits under combined heat and nitrogen-deficient stress in an Aegilops tauschii-derived wheat population
Source: Front Plant Sci. 2025 Jul 16;16:1621916. doi: 10.3389/fpls.2025.1621916 (PMC12307305; doi:10.3389/fpls.2025.1621916)

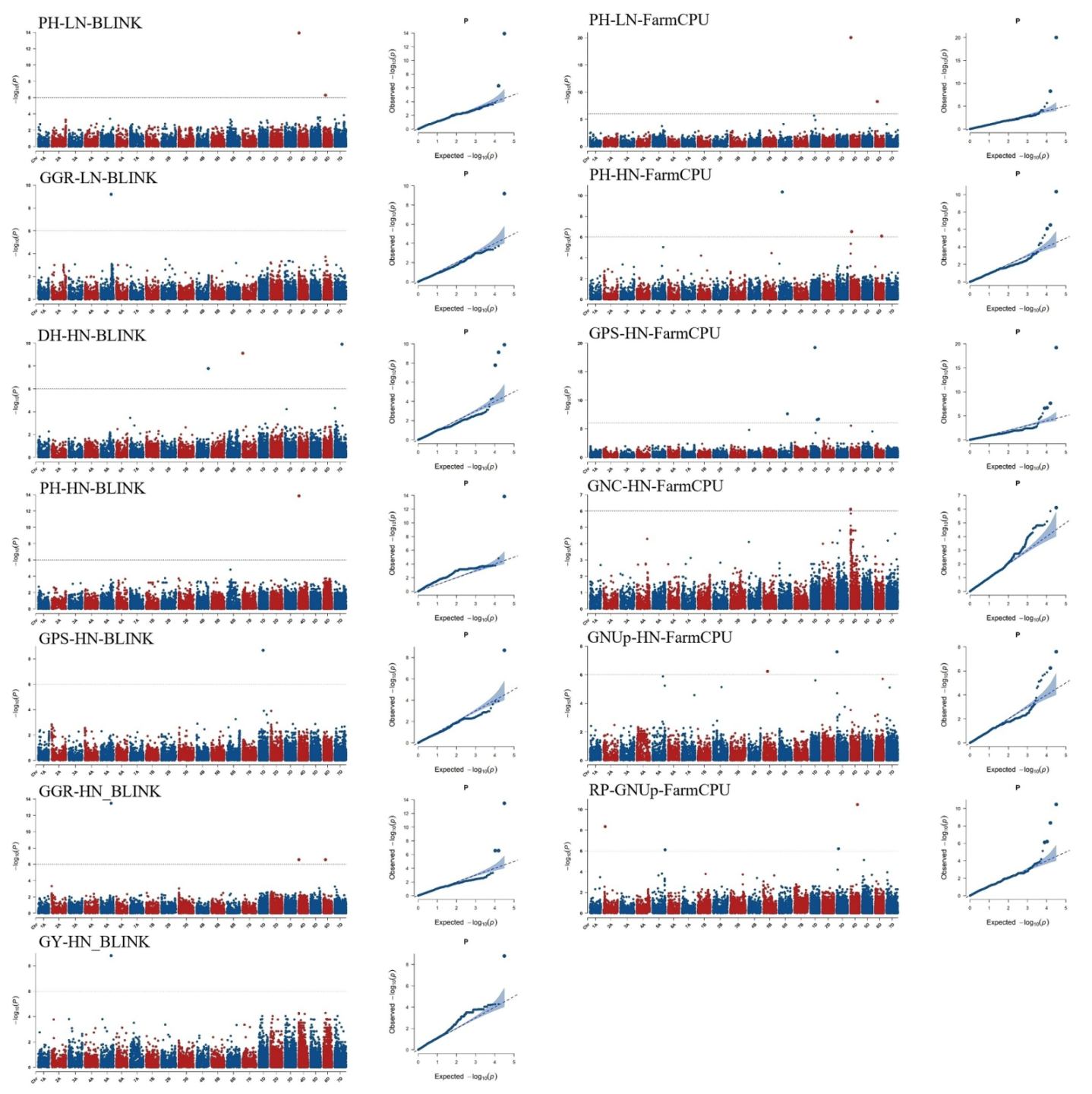

Supplement: Supplementary Figure 1 — GWAS of agronomic, physiological, and NUE-related traits of wheat multiple synthetic derivative (MSD) under heat stress-high N (HS-HN) and heat stress-low N (HS-LN) conditions using Manhattan plot and Q鵬 plot. [file Image1.jpg]

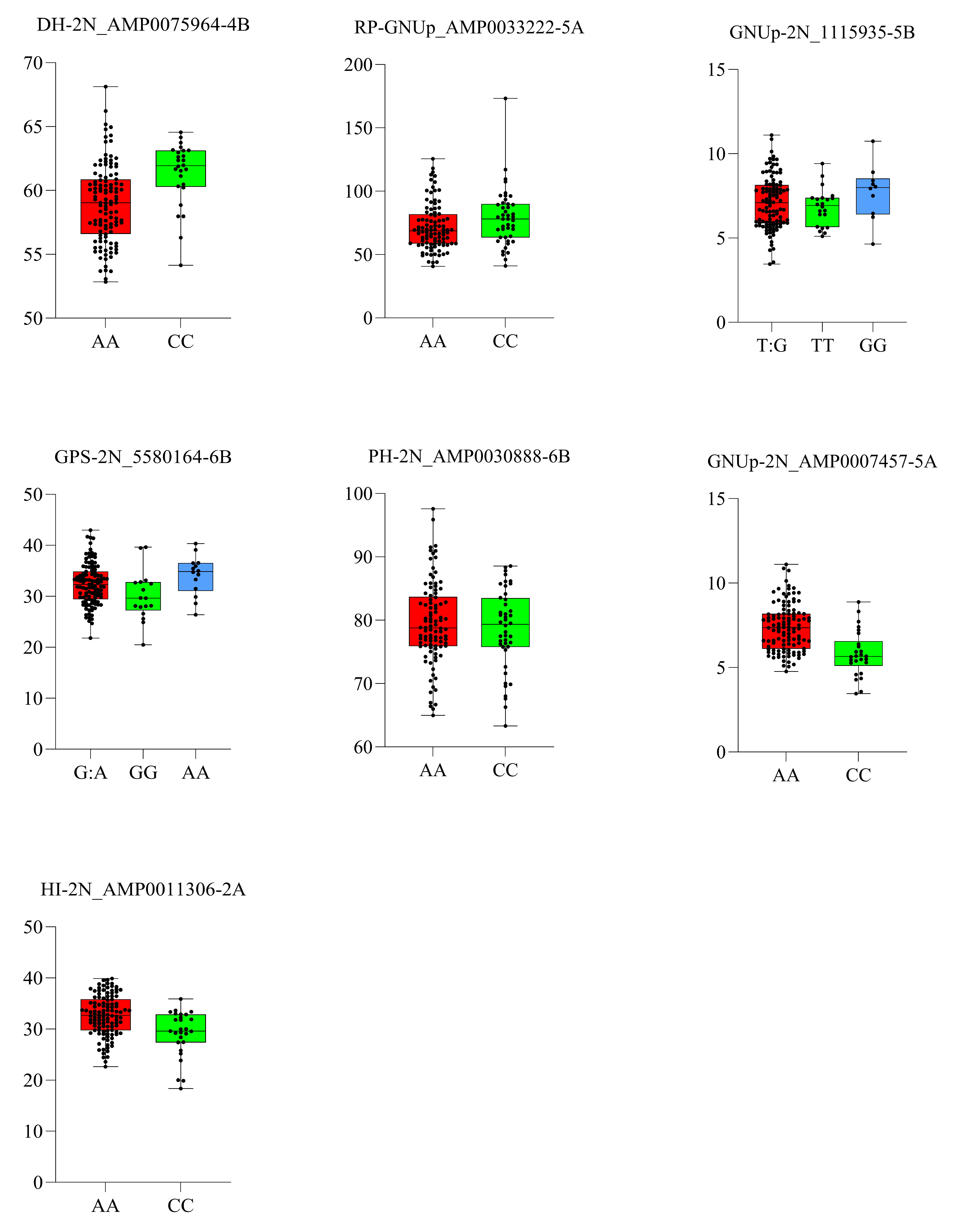

Supplement: Supplementary Figure 2 — Allelic effect of durum (Langdon) allele. The lines with the durum (Langdon) allele are marked in red, whereas those containing Ae. tauschii are indicated in green. [file Image2.tif]
